# Dyes as Photoinitiators or Photosensitizers of Polymerization Reactions

**DOI:** 10.3390/ma3125130

**Published:** 2010-12-02

**Authors:** Jean-Pierre Fouassier, Fabrice Morlet-Savary, Jacques Lalevée, Xavier Allonas, Christian Ley

**Affiliations:** Department of Photochemistry, CNRS, University of Haute Alsace, ENSCMu, 3 rue Alfred Werner, 68093 Mulhouse, Cedex, France

**Keywords:** dyes, photoinitiators, photosensitizers, photopolymerization

## Abstract

A short but up-to-date review on the role of dyes in the photoinitiation processes of polymerization reactions is presented. Radical and cationic reactions are largely encountered in the radiation curing and the imaging areas for use in traditional coating applications as well as in high tech sectors such as nanofabrication, computer-to-plate processing, laser direct imaging, manufacture of optical elements, *etc.* Recent promising developments concerned with the introduction of the silyl radical chemistry that enhances the polymerization efficiency are also discussed.

## 1. Introduction 

Radical and cationic photopolymerization reactions are largely encountered in many industrial applications within the radiation curing and imaging areas. Upon light exposure, a liquid monomer/oligomer matrix leads to either a coating or an image through a chain reaction. As known, this is still extensively growing in traditional industrial sectors related to UV Curing (flooring, packaging, release coatings, powder coatings, wood and medium density fiber (MDF) panels, automotive items, optical fibers, pipe lining, adhesives, graphic arts, dentistry and medicine, *etc*.). On the other hand, the imaging area appears today as a fantastic field of development for new applications in high-tech sectors: microelectronics, Laser Direct Imaging (LDI) technology, Computer-To-Plate processing, 3D modeling, holographic recording and information storage, manufacture of optical elements, design of structured materials on the nanoscale size.

One key factor in photopolymerization reactions is concerned with the photoinitiating systems (PIS), which allow the starting resin formulation to absorb the light and to create reactive species that are further able to initiate the polymerization. A PIS for free radical photopolymerization (FRP) can consist in (i) a photoinitiator (PI; e.g., suitable cleavable ketones), (ii) a photoinitiator (e.g., a ketone) and a co-initiator (coI; e.g., an amine, a thiol, a silane, *etc*.), (iii) a photosensitizer (PS) and a PI or (iv) more complex associations such as PS/PI/coI/RS (where RS is a molecule that improves the efficiency through additional by-side reactions). Basically, the created radicals initiate the polymerization (see e.g., (1a)). In cationic photopolymerization (CP), the PIS is based on a PI (e.g., an onium salt) or a PS/PI couple (1b) (C^+^ in reaction 1b represents the initiating cation); in free radical promoted cationic photopolymerization FRPCP (1c), a radical is produced (e.g., from a usual radical photoinitiator) and then oxidized by an onium salt to form a cation suitable for the opening of the cationic monomer ring. Little is known in anionic photopolymerization.

(1a) $$PIS\;\;\;+\;\;\;h\nu \;\;\;\to \to \to \;\;\;R^{●}\;\;\;\;\to \;\;\;Polymer$$

(1b) $$PIS\;\;\;+\;\;\;h\nu \;\;\;\to \to \to \;\;\;C^{+}\;\;\;\;\to \;\;\;Polymer$$

(1c) $$PIS\;\;\;+\;\;\;h\nu \;\;\;\to \to \to \;\;\;R^{●}\;\;\;\;\to \;\;\;C^{+}\;\;\;\;\to \;\;\;Polymer$$

Most PIs for FRP, CP and FRPCP absorb in the UV wavelength range and directly lead, e.g., (2a), upon excitation, to the desired initiating species (R^●^ or C^+^). Applications requiring the use of visible lights delivered by lamps (Hg lamp, Xe lamp, Hg-Xe lamp, doped Hg lamp, *etc*.), LED, sun or lasers (CW lasers, pulsed lasers, laser diodes, *etc*.) have largely induced strong efforts to (i) search for colored molecules (this has been done for a long time and, among them, dyes represent an interesting class of PI), (ii) design new PIs with red-shifted absorption (which requires much expensive or time-consuming work) and (iii) find well selected PS structures which have to absorb the light and transfer their excitation to a suitable and conventional PI (e.g., 2b, 2c in FRP). These PSs obviously include dyes, but also a lot of other colored molecules that are not, strictly speaking a dye (“a substance used to color materials”); the term “dye” is, however, very often used in a broader sense: in the following, it will refer to any visible light photosensitive PI or PS. 

(2a) $$PI\;\;\;+\;\;\;h\nu \;\;\;(UV\;light)\;\;\;\to \;\;\;PI*\;\;\;\to \;\;\;R^{●}$$

(2b) $$PS\;\;\;+\;\;\;h\nu \;\;\;(visible\;light)\;\;\;\to \;\;\;PS*\;\;\;\to \;\;\;PI*\;\;\;\;\to \;\;\;R^{●}$$

(2c) $$PS\;\;\;+\;\;\;h\nu \;\;\;(visible\;light)\;\;\;\to \;\;\;PS*\;\;\;\to \;\;\;{PS}^{●-}\;\;\;+\;\;\;{PI}^{●+}\;\;\;\;\;\to \;\;\;R^{\prime ●}$$

Here we will give a short but general presentation of dye photosensitized polymerization reactions together with some examples of applications. Many references on UV and visible light curing can be found in previous books [1,2,3,4,5,6,7,8,9,10,11,12,13,14,15,16,17,18,19,20,21,22,23,24] and review chapters [25,26,27,28,29,30,31,32,33,34,35,36,37,38,39,40,41,42] even if most of them are also devoted to other topics. Some promising recent and new developments based on the silyl chemistry will be provided [33,34,35,36].

## 2. Overview of the Available Dye Based Radical Photoinitiators

Dye based PIs can be operative in different photoinitiating systems; review papers and original research papers describe the currently available systems as well as the mechanisms involved in the excited states.

(i) one-component (or Type I) systems where radicals are readily produced through a homolytic bond cleavage (3a),
  (3a) $$PI\;\;\;+\;\;\;h\nu \;\;\;\to \;\;\;{R_{1}}^{●}\;\;\;\;+\;\;\;\;{R_{2}}^{●}$$
(ii) two-component (Type II) systems that involve energy (2b) or electron (2c) transfer in PS/PI combination or electron/proton or direct hydrogen transfer in PI/coI where coI is a hydrogen donating compound AH (3b),
  (3b) $$PI\;\;\;+\;\;\;AH\;\;\;+\;\;\;h\nu \;\;\;\to \;\;\;{PIH}^{●}\;\;\;+\;\;\;A^{●}\;\;\;\;\;\to \;\;\;Polymer$$
(iii) multi-component photoinitiating systems containing three or more compounds and exhibiting a much more complex mechanism, e.g. in (3b-c) in the case of a PI/AH/B system (where it very often appears that A^●^ is the initiating radical whereas PIH^●^ is a terminating radical of the growing polymer chain; B is an additive being able to scavenge the detrimental radicals).
  (3c) $${PIH}^{●}\;\;\;+\;\;\;B\;\;\;\;\;\to \;\;\;{PIH}^{+}\;\;\;+\;\;\;A^{●-}\;\;\;\;\;\to \;\;\;PI\;\;\;+\;\;\;H^{+}\;\;\;+\;\;\;A^{●-}$$

### 2.1. One-component photoinitiating systems 

Examples of one-component photoinitiating systems include:

(i) bisacylphosphine oxides (absorbing up to 480 nm and working through a C-P cleavage);
(ii) organoborates (composed of a borate anion and a visible light absorbing counter cation which allows to tune the absorption);
(iii) organometallic compounds (the most widely used derivatives are the titanocenes which exhibit an excellent absorption around 500 nm);
(iv) metal carbonyl derivatives centered on Mn, Fe, Mo, Cr, Os, Re, Ru;
(v) metal salts and metallic salt complexes based on Co, Fe, Ni, Cu, Cr;
(vi) light absorbing amines;
(vii) difunctional compounds containing two cleavable moieties (which lead to a light absorption better than that of the parent monofunctional compound);
(viii) mono-and di-benzoyl germanes (that absorb around 400 nm);
(ix) bis germyl ketones (the absorption at the Ar laser line is quite good);
(x) group 14 di-metal compounds;
(xi) new transition metal carbonyls.

### 2.2. Two-component photoinitiating systems

Many colored molecules involved in two-component photoinitiating systems have been reported (most of them work through electron/proton transfer with an amine):

(i) carefully selected α-diketones (which allow an excellent efficiency in the near UV-visible region for the photopolymerization of clear thick molded objects or coatings under visible light and sun exposure);
(ii) substituted benzophenone, camphorquinone or thioxanthone and related amino (or thiol) derivatives and thioxanthone-fluorenes;
(iii) various ketone families such as anthraquinone, fluorenone, flavone, anthrone, quinone, naphtacenequinone, quinoline, benzodioxinone;
(iv) xanthenic dyes…, thiazines (methylene blue…), acridines, N-methylacridone, phenosafranines, thiopyronines, riboflavines, phenoxazines, pyrromethenes, polymethines, fluorones, squarylium, julolidine dyes, phenoxazones, quinolinones, phtalocyanines, benzopyranones, rhodanines, crystal violet/benzofuranone derivatives, dimethyl aminostyryl benzothiazolinium iodides. The mechanisms involved in the different systems and the various encountered or promising applications have been reported;
(v) bis-arylimidazole derivatives (for example, Cl-bis-imidazole derivatives HABI are largely used in the laser imaging area; because of the low bond energy between the two imidazoyl moieties, a very fast cleavage occurs leading to two lophyl radicals which then react with electron/hydrogen donors such as mercaptans or N-phenyl glycine);
(vi) coumarin and ketocoumarin derivatives;
(vii) pyrylium and thiopyrylium salts (that can decompose peresters; addition of a diphenyl iodonium salt or a bromo compound such as CBr_4_ to a thiopyrylium salt leads to the generation of radicals);
(viii) ketone/ketone based systems (a typical energy transfer is encountered between a thioxanthone derivative and a morpholino ketone derivative or between a mercaptothioxanthone and a bis phosphineoxide derivative);
(ix) ketone/cleavable compounds (hydroxamic esters, thiohydroxamic esters, *etc.*);
(x) organometallic compound/ketone based systems (e.g., in ruthenium tris bipyridine/morpholinoketone);
(xi) photosensitizer linked photoinitiator or co-initiator based systems (e.g., a dye linked to a triazine moiety or eosin linked to a O-acyloxime);
(xii) miscellaneous systems (xanthene/thiol, carbazole/triazine, photosensitizer/ketone, *etc.*)

### 2.3. Multi-component photoinitiating systems

Multi-component photoinitiating systems involve complex combinations of well selected compounds and are very often used in the design of high speed photopolymers. The mechanisms are rather complex but a clever mixture of suitable compounds allows an improvement in the performance of the PISs and their ability to work in well defined conditions required by the applications. Examples are encountered in:

(i) ketone (e.g., isopropylthioxanthone)/amine/bromo compound or onium salt;
(ii) ketocoumarin/amine/onium salt;
(iii) eosin/amine/onium salt, bromo compound, THF or keto-oxime derivative PDO; methylene blue/amine/onium salt;
(iv) dye (e.g., merocyanine, safranine)/amine/bromo compound or onium salt;
(v) thioxanthene dye/amine/onium salt or bromo compound;
(vi) acridinium cation/dihydropyridine/onium salt;
(vii) ketone/amine/maleimide or maleic anhydride;
(viii) ketone (isopropylthioxanthone/benzophenone sulfonylketone/amine);
(ix) dye/amine/triazine derivatives;
(x) dye (crystal violet, phenosafranine, methylene blue, thiopyronine/amine/ketone) (acetophenone, benzophenone, thioxanthone, 4,4’-bis-dimethylamino benzophenone);
(xi) (keto)coumarin/amine/ferrocenium salt;
(xii) coumarin, ketocoumarin or titanocene derivative/ bis-aryl imidazole Cl-HABI/mercaptobenzoxazole;
(xiii) dye (methylene blue)/amine/cobalt salts;
(xiv) dye (Rose Bengal, eosin)/ferrocenium salt/amine/hydroperoxide;
(xv) coumarin or diethyl amino benzophenone/amine/bis-aryl imidazole/onium salt, pyrromethene/amine/triazine, miscellaneous systems (based on ruthenium derivatives, gold containing molecule, *etc*.).

## 3. Overview of the Available Dye Based Cationic Photoinitiating Systems

### 3.1. Colored onium salts

Useful cationic photoinitiators generally belong to three main classes: diazonium salts, onium salts and organometallic complexes. Iodonium and sulfonium salts absorb in the near UV and represent the main and largely used class of cationic photoinitiators in the radiation curing area. Adequate structural modifications give rise to a red-shifted and enhanced absorption. Alkyl phenyl (9-phenyl thioanthracenyl)-10-sulfonium salts and arylbenzylmethylsulfonium derivatives are sensitive up to 500 nm. The decomposition process is now well established and involves either a heterolytic or homolytic cleavage of the C-I or C-S bond that forms e.g., a singlet phenyliodide/phenyl cation pair or a phenyliodinium cation/phenyl radical, respectively. Then, in-cage or/and an out-of-cage process arises. In any case, a strong acid is generated and initiates the cationic polymerization (CP). The highly sensitive and potentially low cost acylsulfonium salts containing aromatic moieties allows the possibility of a long wavelength excitation. N-alkoxy pyridinium salts also allow efficient photosensitized cationic polymerization reactions.

### 3.2. Organometallic complexes

Organometallic complexes are sensitive in the visible part of the spectrum, e.g., dicarbonyl chelates of IIIa, IVa, Va group elements, ferrocene, zirconocene, organoaluminium complexes, tri-carbonyl iron complexes, various inorganic complexes and organo-metallic transition-metal compounds (metal carbonyl, alkylcyclopentadienyl iron dicarbonyl, *etc.*), non-transition metal complexes (diketone chelates of boron or silicium), rhodium catalyst derivatives, dicobaltoctacarbonyl derivatives, triaryl (1-pyrenyl) bismuthonium salts, cyclopentadienyl arene complexes, bis arene ferrocenium salt derivatives (η^6^−cumene).

### 3.3. Dye/onium salt systems

The possible sensitized decomposition of onium salts On+ in the presence of a photosensitizer (PS) was shown to occur through (i) energy transfer (only with a ketone exhibiting a high lying triplet state) or (ii) electron transfer with hydrocarbons (anthracene, pyrene, perylene, carbazole, *etc.*), phenothiazine derivatives, dyes, ketones (camphorquinone, benzil, anthraquinone, thioxanthone, *etc.*). Anthracene bound sulfonium salts appeared as highly efficient photoinitiators. The electron transfer reactions occur both in the singlet and the triplet states of dyes such as xanthene, thioxanthene and merocyanine, acridone, flavin and acridine derivatives, tetrabenzoporphyrine, curcumin. Aromatic amines, manganese decacarbonyl/alkyl halides, and dithienothiophene act as good electron donors. New recent and promising developments have been published. 

Photosensitization is also feasible through an electron transfer between a radical that can be generated from a usual radical photoinitiator and the iodonium salt: this way is referred to free radical promoted cationic photopolymerization (FRPCP). This mechanism was originally observed with radicals formed through a fast α-cleavage process from the short-lived triplet state of benzoin ethers, dialkoxyacetophenones or benzoyl phosphine oxides; the ketyl radicals produced e.g., in benzophenone/dimethylaniline are also efficient. This reaction was also achieved with other salts (allylic sulfonium salts, phosphonium salts, polysilanes/pyridinium salts, polysilanes/pyridinium salts/benzaldehyde, *etc.*).

Another photosensitizing method is concerned with the addition/fragmentation reaction involving a radical source and a cationic salt containing an allylic group (these two species can also be linked in a single molecule): a careful selection of the radical source allows the absorption to be tuned to higher wavelengths. 

### 3.4. Photoacids PAG

Photoacid generators (PAG) are encountered in the imaging area. They can involve ionic systems (such as the onium salts) or non-ionic systems. The non-ionic photoacids generate sulfinic acid, sulfonic acid, carboxylic acid, phosphoric acid. They involve several kinds of structures e.g., (i) iminosulfonates (the sulfonic acid is photochemically produced in a first step; in a second step, a thermal process favors the crosslinking reactions of e.g., epoxides, a negative image will thus be obtained under solvent development); (ii) esters of sulfonic or carboxilic acids; (iii) arene sulfonate derivatives; (iv) hydroxyimide sulfonates; (v) oxime sulfonates; (vi) sulfone derivatives; (vii) triaryl phosphate derivatives; (viii) naphtalimides; their photosensitized decomposition under visible lights is, however, usually rather difficult. The photochemistry, photophysics, photopolymerization activity and photoimaging applications of naphthalimides in the presence of photosensitizers has been largely explored.

Most of these systems are used in organic high speed photopolymers that can be excited by visible laser lights. Sensitizer/PAG systems containing dispersed photopolymers have been developed for micron-scale photolithography. A chemistry based on a photochemical event that requires one photon to produce one acid has obviously a limited efficiency in photoresist applications. The chemical amplification process where one photon is able to indirectly induce several reactions was evidenced in negative photoresists but also in positive photoresists (e.g., depolymerization of polyphthaldehyde or deprotection of the acidic function of a polymer that will become soluble in an alkali; a post baking treatment favors the acid diffusion and the chemical amplification; this acid catalyzed reaction produces a positive relief image).

## 4. Dye Based Anionic Photoinitiators and Photobase Generators (PBG)

Compared to radical and cationic photoinitiating systems, anionic PIs and PBGs are considerably less encountered. Available structures that are able to liberate an anion or a base upon light exposure and are usable in photocrosslinking reactions have been reported in several papers. They involve oxime esters, carbamates, 9-fluorenyl carbamates, ferrocenophanes, ammonium tetraorganyl borate salts, amidine based N-benzylated structures, aromatic formamides, cobalt-amine complexes, amine-imides, aminoketones, phosphazene bases, dye leuconitriles. Except for very few systems, the absorption usually occurs in the UV. The photosensitized decomposition of some structures has been observed with benzophenone, thioxanthone or naphtoquinone. 

## 5. Recent Developments of Silane Based System for FRP, CP and FRPCP upon Visible Light Excitation under Air

Oxygen is a well known detrimental agent in any reaction involving a radical. In FRP, a serious drawback concerns the oxygen inhibition. The same holds true in FRPCP. According to their lifetimes, excited states are more or less quenched by O_2_. The oxygen/radical interaction is a nearly diffusion controlled reaction. Both the initiating and propagating radicals are scavenged by O_2_; they lead to highly stable peroxyl radicals. 

The polymerization only starts (inhibition period) when oxygen is consumed: (i) in highly viscous or thick samples, the re-oxygenation process is quite slow and the inhibition time is short, (ii) on the opposite, in very low viscosity or thin samples, the re-oxygenation remains efficient, (iii) in addition, the viscosity change can strongly affect the reaction rate constants under air and lead to a leveling of their values, (iv) when the light intensity is reduced, the amount of initiating radicals becomes lower and most of them are thus scavenged by O_2_. The importance of the oxygen inhibition effect is strongly dependent on the experimental conditions.

Previous experiments using polysilanes in FRPCP were reported, the silyl radicals being generated by the cleavage of the Si-Si bonds under light irradiation. However, this method suffers from two main drawbacks: (i) a low cleavage quantum yield is usually observed and (ii) some other by-side reactions are competitive to the silyl radical formation. 

To rule out the above problems, a different approach where the formation of silyl radicals occurs through a hydrogen abstraction process from R_3_SiH was recently proposed and found applications in FRP and FRPCP. This interesting development opens new opportunities and appears as really versatile: it consists in the incorporation of silanes (and to a lesser extent boranes or stannanes) into usual PIS combinations or to use a silane with a suitable absorbing hydrogen acceptor. The introduction of a silyl moiety on a given PI or PS backbone also allows design of efficient new PISs but this way is more expensive. In any cases, silyl radicals are created. 

A large tuning of the absorption range has been achieved using BAPO (or TPO)/silane, ITX/silane, Eosin/silane, thiopyrylium salt/silane, titanocene derivative/silane, thiopyrylium salt/silane/onium salt, thiopyrylium salt/disilane, ruthenium/silane/onium salt, *etc*. Some typical examples outlining the performance of organosilanes (tris(trimethylsilyl)silane TTMSS and tetrakis(dimethylamido)silane TDMAS) containing photoinitiating systems will be briefly presented.

### 5.1. Silyl radicals for FRPCP processes

**Scheme 1:**
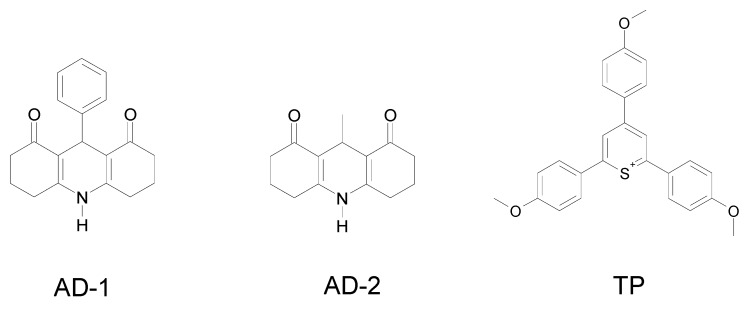


2,4,6(4-methoxyphenyl)thiopyrylium (TP), camphorquinone, Eosin-Y and two derivatives of acridinediones (AD-1 and AD-2) are used as representative photosensitive systems exhibiting a good light absorption at λ > 400 nm (Scheme 1).

**Figure 1:**
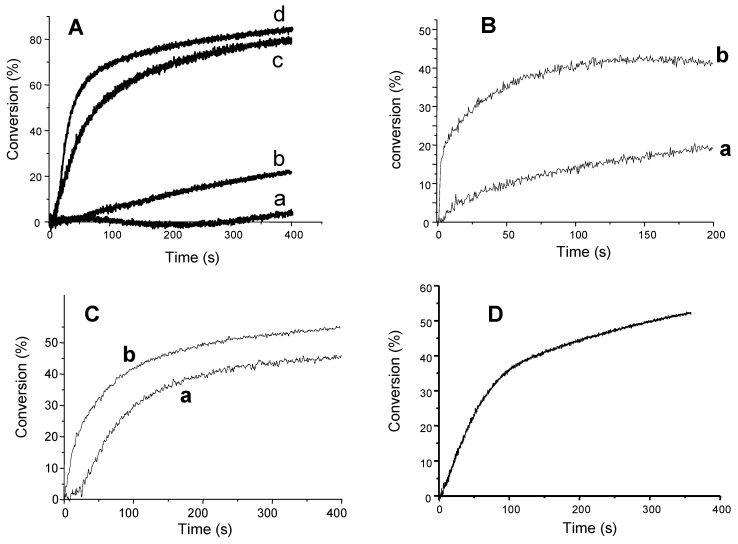
Photopolymerization profiles of EPOX under air. Upon Xenon lamp irradiation (λ > 390 nm) in the presence of (**A**) a: **AD-1**/Ph_2_I^+^ (3%/2% w/w); b: **AD-2**/Ph_2_I^+^ (3%/2% w/w); c: **AD-2**/TTMSS/Ph_2_I^+^ (3%/3%/2% w/w); d: **AD-1**/TTMSS/Ph_2_I^+^ (3%/3%/2% w/w); (**B**) a: **TP**/Ph_2_I^+^ (0.1%/1% w/w) and b: **TP**/TTMSS/Ph_2_I^+^ (0.1%/10%/1% w/w); (**C**) a: camphorquinone/ethyldimethylaminobenzoate/Ph_2_I^+^ (3%/3%/1% w/w); b: camphorquinone/TTMSS/Ph_2_I^+^ (3%/3%/1% w/w); (**D**) Eosin-Y/TTMSS/Ph_2_I^+^ (0.05%/3%/1% w/w); in the absence of TTMSS, no polymerization is observed.

Their ability to initiate the cationic polymerization of an epoxy monomer ((3,4-epoxycyclohexane)methyl 3,4-epoxycyclohexylcarboxylate EPOX, Uvacure 1500 from Cytec) is shown in Figure 1. The polymerization profiles are much better upon addition of TTMSS thereby demonstrating the huge effect of the presence of a silane *i.e*., for Eosin-Y/Ph_2_I^+^, photopolymerization does not occur but an efficient process is found when using Eosin-Y/TTMSS/Ph_2_I^+^. Such a situation is also noted for AD-1, AD-2 and TP. Interestingly with camphorquinone, TTMSS appears better than an amine such as ethyldimethylaminobenzoate (Figure 1C curve a *vs*. curve b). A thermal degradation involving the iodonium salt prevents the use of TDMAS. 

The good performance achieved with the organosilanes is ascribed to the formation of silyl radicals by a hydrogen abstraction reaction (5) between the silane and the phenyl radicals generated through the sensitized decomposition of the iodonium salt (4).

(4) $$*PS+{Ph}_{2}I^{+}\to {PS}^{●+}+\text{Ph-I}+{Ph}^{●}$$

(5) $${Ph}^{●}+R_{3}\text{Si-H}\to Ph-H+R_{3}{Si}^{●}$$

The silyl radicals are easily oxidized by the iodonium salt leading to silylium cations which are efficient initiating species for the ring opening polymerization of epoxy monomers (6–7).

(6) $$R_{3}{Si}^{●}+{Ph}_{2}I^{+}\to R_{3}{Si}^{+}+\text{Ph-I}+{Ph}^{●}$$

(7) $$R_{3}{Si}^{+}+M\to \to \to polymer$$

### 5.2. Silyl radicals for FRP processes

**Figure 2:**
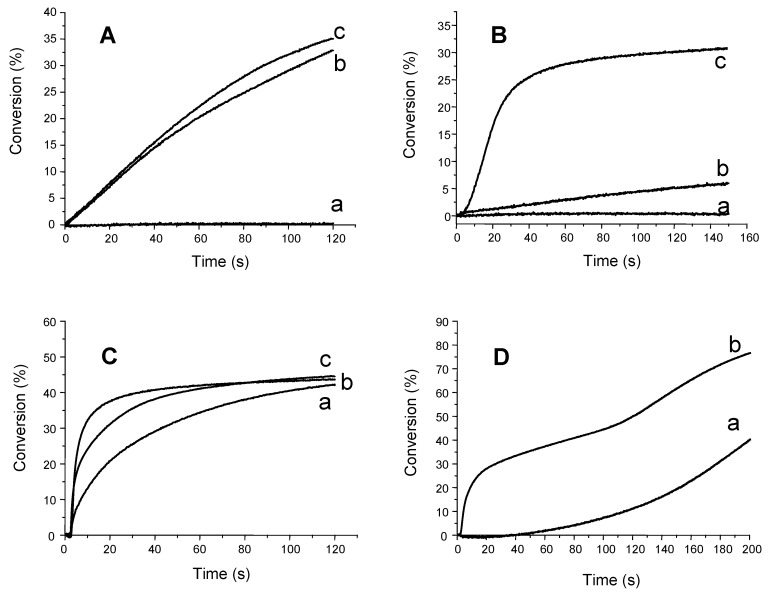
Photopolymerization profiles of TMPTA under air. Upon Xenon lamp irradiation (λ > 390 nm) in the presence of (**A**) a: Eosin-Y/ethyldimethylaminobenzoate (0.05%/3% w/w); b: Eosin-Y/TTMSS (0.05%/3% w/w); c: Eosin-Y/TDMAS (0.05%/3% w/w); (**B**) a: ITX (1% w/w); b: ITX/Ph_2_I^+^ (1%/1% w/w); c: ITX/TTMSS (1%/3% w/w); (**C**) a: camphorquinone/ethyldimethylaminobenzoate (3%/3% w/w); b: camphorquinone/TTMSS (3%/3% w/w); c: camphorquinone/TDMAS (3%/3% w/w); (**D**) a: **AD-2** (3% w/w); b: **AD-2**/TTMSS (3%/3% w/w).

The silyl radicals are characterized by very high addition rate constants to double bonds and can efficiently initiate FRP of acrylate monomers. The silyls are usually also generated by a hydrogen abstraction reaction. Typical photopolymerization profiles of trimethylolpropane triacrylate (TMPTA from Cytec) in the presence of dye (isopropylthioxanthone, camphorquinone, Eosin-Y and acridinedione AD-2)/silane (TTMSS or TDMAS) are displayed in Figure 2. Silanes are often better than ethyldimethylaminobenzoate (EDB), which is a well known reference radical co-initiator.

The ability of organosilanes to overcome the oxygen inhibition effect is related to the conversion of stable peroxyl radicals generated under air by the scavenging of initiating or propagating radicals into new reactive silyl radicals (8); this results in a consumption of the Si-H function during the polymerization.

(8) $${ROO}^{●}+R_{3}\text{Si-H}\to \text{ROO-H}+R_{3}{Si}^{●}$$

## 6. Conclusions

All these photoinitiating systems can be encountered in a large variety of reactions in the radiation curing area (film photopolymerization and crosslinking of acrylates and epoxides, thiol-ene photopolymerization, photopolymerization of water borne light curable systems, photopolymerization of powder formulations, controlled photopolymerization (RAFT, ATRP, NMP), charge transfer photopolymerization, dual cure photopolymerization, *etc.*) and the Imaging and Laser imaging area (writing of complex relief patterns for the manufacture of microcircuits and computer-to-plate CTP systems, three dimensional machining, 3D microscale structuration for micro-electromechanical, micro-optics and microfluidic applications, manufacture of gratings, microlenses or waveguides, holographic recording and storage media, *etc.*). Recent promising developments in applications where severe conditions are imposed (e.g., in sunlight induced polymerization) or new monochromatic light sources (LED, laser diodes) are used outline the crucial role of the dye in the formulation. In the same way, the careful choice of the dye based photoinitiating system and the design of two-photon sensitive dyes for laser induced polymerization opens up interesting routes in the nanotechnology field. Fortunately, as shown in this short review, many dye based radical and cationic photosensitive systems are already available.
